# The contribution of minimally invasive tissue sampling compared to antemortem-derived cause of death determination among inpatient child deaths: the minimally invasive tissue sampling in Malawi study

**DOI:** 10.7189/jogh.15.04210

**Published:** 2025-08-04

**Authors:** Wieger P Voskuijl, Dennis Chasweka, Sarah Lawrence, Daniella Brals, Steve Kamiza, Robert Bandsma, James A Berkley, Emmie Mbale, Charalampos Attipa, Chisomo Eneya, Cornelius Huwa, Stanley Khoswe, Christopher Moxon, Isabel Potani, Jessica L Waller, Maureen H Diaz, Judd Walson, Jaume Ordi, Donna M Denno

**Affiliations:** 1 Amsterdam UMC, University of Amsterdam, Amsterdam Centre for Global Child Health, Emma Children’s Hospital, Amsterdam, the Netherlands; 2 Amsterdam UMC, University of Amsterdam, Department of Global Health, Amsterdam Institute for Global Health and Development, Amsterdam, the Netherlands; 3 Department of Paediatrics and Child Health, Kamuzu University of Health Sciences, Blantyre, Malawi; 4 The Childhood Acute Illness and Nutrition (CHAIN) Network, Nairobi, Kenya; 5 Department of Pathology, Kamuzu University of Health Sciences, Blantyre, Malawi; 6 KEMRI/Wellcome Trust Research Programme, Kilifi, Kenya; 7 Centre for Tropical Medicine and Global Health, Nuffield Department of Medicine, University of Oxford, UK; 8 The Royal (Dick) School of Veterinary Studies and The Roslin Institute, University of Edinburgh, Edinburgh, Scotland, UK; 9 Malawi-Liverpool-Wellcome Programme, Kamuzu University of Health Sciences, Blantyre, Malawi; 10 Wellcome Centre for Integrative Parasitology, School of Infection and Immunity, University of Glasgow, Glasgow, Scotland, UK; 11 School of Infection and Immunity, University of Glasgow, Glasgow, Scotland, UK; 12 Blantyre Malaria Project, Kamuzu University of Health Sciences, Blantyre, Malawi; 13 Malawi-Liverpool-Wellcome Programme, Kamuzu University of Health Sciences, Blantyre, Malawi; 14 National Centre for Immunisation and Respiratory Diseases, Centres for Disease Control and Prevention, Atlanta, Georgia, USA; 15 Bloomberg School of Public Health, John Hopkins University, Baltimore, Maryland, USA; 16 ISGlobal-Hospital Clínic, Universitat de Barcelona, Barcelona, Spain; 17 Department of Pediatrics, University of Washington School of Medicine, Seattle, Washington, USA; 18 Department of Global Health, University of Washington School of Public Health, Seattle, Washington, USA; 19 Hospital for Sick Children, Translational Medicine Program and Centre for Global Child Health, Toronto, Canada

## Abstract

**Background:**

Improved causes of death (CoD) understanding in low- and middle-income countries is needed to reduce child mortality. Compared to full autopsy, minimally invasive tissue sampling (MITS), using transcutaneous needle sampling, is a feasible, socially acceptable, and validated method. We aimed to quantify the additional contribution of MITS to CoD attribution based on clinical records and inpatient research data with intensive patient characterisation.

**Methods:**

We enrolled children aged seven days to 59 months who died while on admission for acute illness and/or severe malnutrition to Queen Elizabeth Central Hospital in Blantyre, Malawi. Standard MITS procedures included histologic, immunohistochemical, and microbiologic testing. Phase 1 CoD determination was based on medical records alone, Phase 2 also included research data, and Phase 3 included all data, including from MITS.

**Results:**

We enrolled 29 children. Based on clinical notes alone (Phase 1), we identified 60 causal and 39 contributing conditions. Of the 45 (45%) infectious conditions, pathogens were identified in 15 (33%). Only one patient’s (3%) CoD was unchanged compared to including all data (Phase 3). Further, we identified 69 new (n = 43) or adjusted (n = 26) diagnoses among 28 cases (97%); the majority were undernutrition-related (n = 22, 32%) or infectious (n = 41, 59%) conditions. Overall, the majority of final Phase 3 conditions were also undernutrition-related (n = 46, 32%) or infectious (n = 61, 43%) and a pathogen was identified in 54 (89%) of the infectious conditions. *Klebsiella pneumoniae* was the most prevalent aetiology in both pneumonia and sepsis.

**Conclusions:**

The addition of MITS to clinical and inpatient research data led to almost all (97%) of cases receiving new and/or refined diagnoses, including microbe identification in infectious conditions. Pathogens not specifically addressed by current clinical guidelines, such as *Klebisiella pneumoniae,* were commonly identified. Our findings support the utility of MITS to understand CoD even after thorough clinical characterisation of children during hospitalisation.

Despite a substantial decline in child mortality over the past decades, the pace of this reduction is not on track to meet the Sustainable Development Goals’ child mortality target [1]. Infectious diseases such as malaria, diarrhoea and pneumonia remain important causes of death, and undernutrition is an underlying contributor to 45% of deaths among children aged <5 years [2]. However, causes of and contributors to child deaths in low- and middle-income countries (LMICs) are often undetermined or imprecisely or incompletely characterised [3]. The gold standard for cause of death (CoD) ascertainment is the complete diagnostic autopsy, but this is rarely performed or feasible in LMICs [4,5]. Verbal autopsy – a structured post-mortem interview with a parent/caregiver – is commonly used, but imprecise in identifying many conditions (*e.g.* malnutrition) and differentiating between others (*e.g.* infection aetiologies) [6].

Postmortem minimally invasive tissue sampling (MITS) uses transcutaneous needle organ and fluid sampling is increasingly being used in research studies in LMICs as a validated, non-disfiguring, and more acceptable alternative to complete diagnostic autopsy [5,7–9]. MITS is often deployed in circumstances where limited antemortem clinical information is available; however, as far as we are aware, no analyses have been conducted showing MITS CoD ascertainment compared to traditional clinical approaches based on medical files or compared to detailed antemortem research data when it is available. Our research group has been engaged in the Childhood Acute Illness and Nutrition (CHAIN) cohort study [10] and other inpatient clinical studies to identify risk factors for mortality among children hospitalised with acute illness and/or undernutrition through extensive biological and social characterisation.

Our aim was to fill the knowledge gap by quantifying the additional contribution of data from MITS to CoD derived from hospital clinical records alone and to CoD derived from clinical records plus detailed research data, among children who died during an inpatient admission for acute illness and/or undernutrition in Malawi. We hypothesised that CoD, as determined by MITS, would add to or differ from clinical CoD determination in 30% of cases and that CoD ascertained from clinical and research data would be minimally different.

## METHODS

### Study population

The MITS in Malawi study (MiM) was conducted at Queen Elizabeth Central Hospital, a national referral and teaching hospital in Blantyre, Malawi. MiM began as a sub-study of the CHAIN cohort study, which aimed to assess pathways to mortality among children hospitalised with acute illness or undernutrition in LMICs [10,11]. Because of lower than anticipated enrolment and case fatality in CHAIN for the current objective, MiM recruitment was expanded to two other Queen Elizabeth Central Hospital-based studies also examining risk factors for paediatric inpatient mortality – one focused on clinical assessments of encephalopathies and the other on metabolic assessments of undernourished children (another CHAIN sub-study) – and to children not enrolled in any research study. MiM included children aged seven days to 59 months who died during hospitalisation for acute illness and/or undernutrition. Exclusion criteria were known terminal illnesses, congenital syndromes, injuries, or surgical conditions.

Recruitment occurred from 20 August 2018 to 9 April 2020. Study staff approached parents/guardians of eligible children after a respectful waiting period following the death. We developed the informed consent process based on prior qualitative research involving key hospital staff and community members (religious leaders and parents of children aged <5 years) in Malawi [9]. We obtained written informed consent. Assistance (*e.g.* with transportation) and grief support were offered to parents/guardians, regardless of participation status.

### Antemortem clinical data collection

We abstracted from medical records history, vital signs, anthropometric measurements, physical examination findings, and clinical laboratory values for all study participants. We prospectively and systematically recorded on study case report forms (CRFs) detailed clinical data and standardised laboratory results (including complete blood cell count, plasma glucose concentration, rapid malaria test, and HIV testing) [12] for children co-enrolled in inpatient studies – henceforth referred to as ‘intensive research studies’ (IRS) data.

### Anthropometric measurements

Two study personnel measured mid-upper arm circumference (MUAC), weight, and length, and where discrepant, measured a third time. We used the average of the two closest measurements. We calculated Z-scores using the World Health Organization (WHO) 2006 standards [13]. We defined severe wasting (‘marasmus’) as MUAC<11.5 cm (among children aged ≥6 months) or a weight-for-length Z score<–3, nutritional oedema (‘kwashiorkor’) by the presence of bilateral pitting oedema, and moderate wasting as MUAC<12.5 cm but ≥11.5 cm (among children aged ≥6 months) or weight-for-length Z score<−2 but≥−3 following WHO and recently published guidelines on anthropometry interpretation within MITS studies [7,14]. We also accepted classification of antemortem severe wasting if antemortem MUAC and length were not recorded but clinical records described visible severe wasting on examination.

### MITS procedures

We adopted procedures from standard protocols [15] and commenced as soon as possible after consent, starting with inspection of the body and postmortem anthropometry. Standard MITS sampling [15] included blood, cerebrospinal fluid (CSF), lung and stool for microbiological analysis and brain, lung, heart, liver, and kidney for histological evaluation. All samples collected for microbiology were either immediately cultured or frozen until molecular analysis. We fixed all tissues collected for histological evaluation in 10% neutral buffered formalin.

### Bacterial culture and molecular testing

We collected blood samples into EDTA containers for molecular testing and paediatric aerobic bottles for culture. We performed blood culture on an automated blood culture system (BacT/Alert bioMérieux, Lyon, France) and organisms were identified by standard phenotypic microbiology methods as described previously [16]. We collected CSF into sterile universal containers, performed a Gram stain, and cultured the samples using conventional methods as previously described [17]. We conducted HIV rapid diagnostic testing (if determined (Abbott) positive, further testing by Unigold (Trinity Biotech); with polymerase chain reaction (PCR) confirmation among children <18 months old) and malaria rapid (Bioline) and microscopy testing when antemortem tests had not been conducted. We extracted total nucleic acid, both DNA and RNA, from nasopharyngeal swabs, lung biopsies, stool (from rectal brushings), whole blood, and CSF specimens. Specimen processing for extraction varied based on the type of specimen being tested, as previously described [15]. Following pre-processing, we extracted all nucleic acid manually using the Qiagen QIAamp fast DNA stool mini kit (Qiagen, Sandton, South Africa) and following the kit protocol from the first ethanol wash through completion, except for treating specimens with ribonucleases. This ensured that we collected both DNA and RNA. We tested the collected nucleic acid for each specimen on custom-developed syndromic TaqMan Array Cards (TAC), a 384-well microfluidic array of real-time PCR assays detecting multiple pathogens simultaneously. We tested nasopharyngeal swabs and lung biopsies on the custom respiratory TAC, stools on the custom enteric TAC, and blood and CSF specimens on the custom blood/CSF TACs, as previously described [18]. The targets encompass detection of 30 bacteria genera, 40 viruses, eight parasites, and three fungi (Table S1 in the **Online Supplementary Document**). Additionally, point-of-care dipstick urinalysis and routine CSF protein concentration and white and red blood cell counts were conducted.

For histological evaluation, we paraffin-embedded all samples, sectioned and stained them with haematoxylin and eosin (HE). One pathologist (SK) read all HE slides and entered results on standardised histology CRFs. The second pathologist (JO) conducted quality control reads for approximately every fifth case. The two pathologists were easily able to resolve rare inter-reader discrepancies by discussion. We performed confirmatory ancillary histochemical (*i.e.* Gram, Ziehl-Neelsen, Periodic acid-Schif, Grocott methenamine) and immunohistochemical stains (for cytomegalovirus, human herpesvirus 8, *Klebsiella pneumoniae, Plasmodium falciparum,* respiratory syncytial virus, and *Treponema pallidum*) in selected cases using the Roche Ventana platform (VENTANA BenchMark Special Stains system, BenchMark ULTRA IHC/ISH System, Roche diagnostics, Rotkreuz, Switzerland). A study pathologist (JO) read them to confirm or exclude specific diagnoses suspected based on HE stain, culture, or TAC results following the methodology described elsewhere [15].

### Determination of cause of death (DeCoDe)

We assigned CoD in three phases. In each phase, we coded diagnoses according to the International Classification of Diseases-10 [19]. To mitigate subjectivity and variability in interpretation, we followed the Child Health and Mortality Prevention Surveillance (CHAMPS) (a large MITS-based paediatric CoD surveillance study) DeCoDe standard operating procedure (SOP) and the recently published guideline on coding undernutrition diagnoses in MITS studies to categorise mortality causes and contributors for each child in each phase [7,20]. We recorded conditions considered in the causal chain in part one of the WHO death certificate and further classified them as immediate, intermediate, or underlying CoD (per WHO standards) [21]. We considered the disease which initiated the chain of events leading to death the ‘underlying CoD’. The ‘intermediate CoD’ refers to any condition(s) leading from the underlying to the ‘immediate CoD’, which is the condition directly leading to death. We recorded conditions deemed as contributors to, but not causes of death, in part two of the WHO death certificate. We additionally followed the CHAMPS DeCoDe SOP to code level of certainty (LOC) (1 = certain, 2 = probable, 3 = likely) for all conditions [20]. The process of assigning LOC to each diagnosis is based on the completeness and specificity of available data substantiating the diagnosis [20].

### DeCoDe phases

Phase 1 and 2 CoD coding excluded any MITS-derived data (*i.e*. only included clinical and intensive research study-derived data, respectively). Two paediatricians with expertise in global child health (DD and WV) independently, and blinded to case number, assigned the causal and contributing condition/s for each case, as well as the LOC for each condition. Phase 1 coding was based on data abstracted from clinical records only. Phase 2 additionally included a review of IRS data from study CRFs for the 10 children who were co-enrolled in inpatient studies. Inter-coder differences were identified and resolved through consensus meetings.

Phase 3 CoD determination was conducted by DeCoDe panel meetings, comprised of the two paediatricians, a paediatric infectious disease specialist (CM), and two pathologists with expertise in MITS (SK and JO). In advance of these meetings, each of the five panellists prepared their part one and part two diagnoses, placement within part one, and LOC assignments based on their review of data from the clinical notes, IRS CRFs (n = 10 children) and all post-mortem evaluations. Each diagnosis, placement in the mortality causal chain or as a contributor, and LOC was discussed until group consensus was reached.

### Statistics

We used descriptive statistics to calculate the prevalence of diagnoses and LOCs in each coding Phase (1 = clinical, 2 = clinical plus IRS, and 3 = clinical, IRS, plus MITS-derived data).

## RESULTS

During the enrolment window, 75 children died on recruitment days and were eligible for MiM, and we approached 58 for consent. Reasons for not seeking consent were related to inability to mobilise a key study member within an appropriate timeframe (n = 8), lack of notification by ward staff (n = 5), or unknown (n = 4). Of 58 caregivers approached, 29 (50%) consented to participation. Reasons for refusal were related to lack of perceived benefit (n = 9), preference to immediately take the body home (n = 7), cultural/religious concerns (n = 5), poor relationship with healthcare staff (n = 2), consenting team unavailable (n = 2), child too young (n = 1), and unknown (n = 3).

The median (MD) participant age was MD = 24 weeks (interquartile range (IQR) = 12–55), 15 (52%) were female, and the length of hospitalisation was MD = 3 days (IQR = 1–6). The 10 patients co-enrolled in inpatient studies were older (MD = 47 weeks, IQR = 26–90), 30% were female, and hospitalisation was longer (MD = 6 days, IQR = 3–9).

### Causes of and contributors to death based on clinical notes (Phase 1)

**Figure 1:**
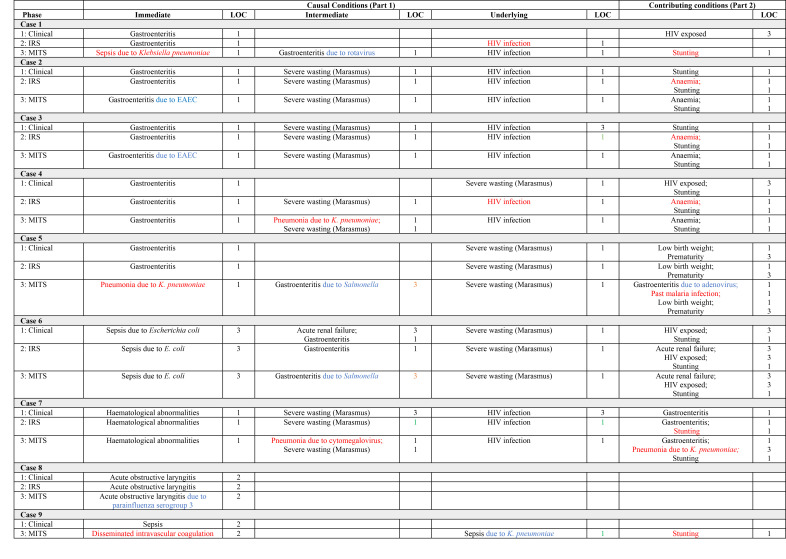
Cases 1–8. Causes of and contributors to death and the level of certainty (LOC) of the diagnoses identified in Phases 1 (clinical notes data), 2 (clinical notes + IRS data), and 3 (clinical notes + IRS + MITS data). Key to colour coding: Red – new diagnosis; Blue – adjusted diagnosis; Green – improved LOC; Orange – reduced LOC. EAEC – enteroaggregative *E. coli*, IRS – Intensive Research Studies, LOC – level of certainty, MITS – minimally invasive tissue sampling.

**Figure 2:**
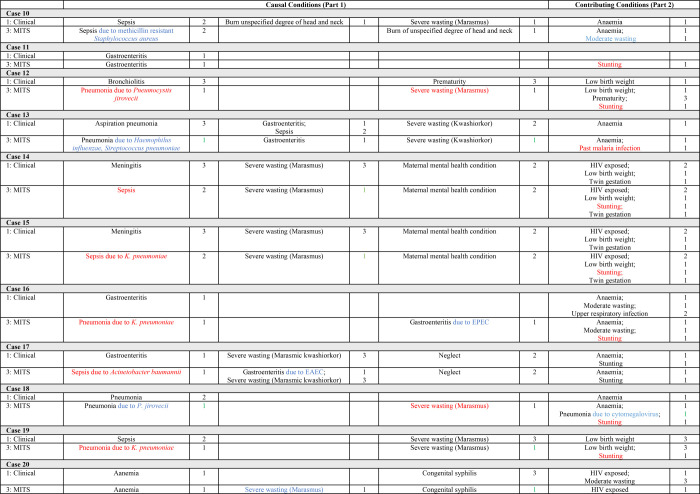
Cases 9–20. Causes of and contributors to death and the level of certainty (LOC) of the diagnoses identified in Phases 1 (clinical notes data), 2 (clinical notes + IRS data), and 3 (clinical notes + IRS + MITS data). Key to colour coding: Red – new diagnosis; Blue – adjusted diagnosis; Green – improved LOC; Orange – reduced LOC. EAEC – enteroaggregative *E. coli*, EPEC – enteropathogenic *E. coli*, IRS – Intensive Research Studies, LOC – level of certainty, MITS – minimally invasive tissue sampling.

**Figure 3:**
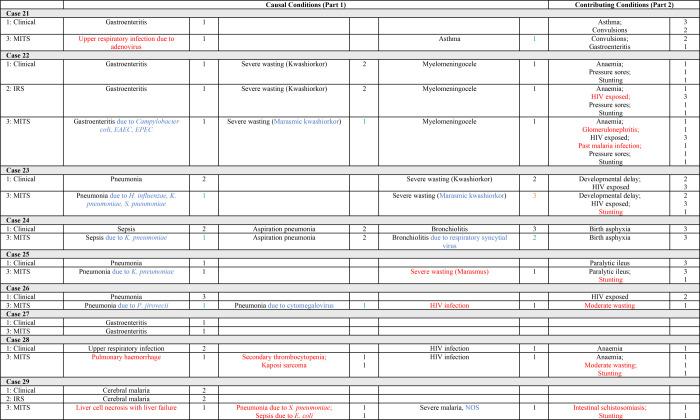
Cases 21–29. Causes of and contributors to death and the level of certainty (LOC) of the diagnoses identified in Phases 1 (clinical notes data), 2 (clinical notes + IRS data), and 3 (clinical notes + IRS + MITS data). Key to colour coding: Red – new diagnosis; Blue – adjusted diagnosis; Green – improved LOC; Orange – reduced LOC. EAEC – enteroaggregative *E. coli*, EPEC – enteropathogenic *E. coli*, IRS – Intensive Research Studies, LOC – level of certainty, MITS – minimally invasive tissue sampling, NOS – not otherwise specified.

Based on the clinical notes review (Phase 1) in the 29 cases, we coded a total of 99 diagnoses. Of these 60 were in the causal chain (whether immediate, intermediate, or underlying) and 39 were contributing conditions (**Figures 1–3**). Further, 27 (27%) were undernutrition diagnoses (low birthweight, stunting, moderate wasting, severe wasting, or nutritional oedema), including 14 (23%) in the causal chain. Moreover, 45 (45%) of all diagnoses were infectious conditions, including 35 (58%) in the causal chain. We identified a pathogen for 15 (33%) of the infectious conditions, primarily related to HIV infection or maternal exposure (n = 12). Pathogens in the causal chain (n = 7) included HIV infection based on testing (n = 4), sepsis attributed to *Escherichia coli* based on blood culture (n = 1), malaria based on a rapid test (n = 1), and congenital syphilis based on clinical appearance (n = 1). All eight infectious contributing conditions were maternal exposure to HIV. Further, six of these were based on maternal history, while two also had test results recorded in the clinical notes.

In 11 of the 29 cases, there was a single CoD (**Figures 1–3**); hence, the immediate CoD was also considered the underlying CoD per WHO death certification guidelines [21]. The most prevalent underlying CoDs among the 29 cases were severe acute malnutrition (n = 7), gastroenteritis (n = 5), HIV infection (n = 4), and respiratory infections (n = 4), including pneumonia (n = 3). Further, 16 (55%) of the underlying CoDs were infectious. Moreover, 26 (90%) of the immediate CoDs were infectious and the most prevalent included gastroenteritis (n = 11), respiratory infections (n = 7), including pneumonia (n = 4), and sepsis (n = 5). Of the 39 conditions contributing to mortality, the most prevalent were maternal HIV exposure (n = 8), anaemia (n = 7), stunting (n = 6), low birth weight (n = 5), and moderate wasting (n = 2).

### Causes of and contributors to death, including IRS data (Phase 2)

For the 10 participants co-enrolled in inpatient studies, 36 diagnoses were identified through clinical notes alone, while we coded 41 when the IRS data was also analysed. The new diagnoses in Phase 2 were anaemia (n = 3), stunting (n = 1), and HIV exposure (n = 1) as contributing conditions (**Figures 1–3**). In addition, we identified two HIV infection diagnoses (originally coded as HIV exposure based on maternal history or antibody test). Anaemia and HIV-related diagnoses were due to systematic complete blood cell count and HIV testing (including PCR if antibody positive among children aged <18 months) per the inpatient research protocols.

### Causes of and contributors to death when including MITS-derived data (Phase 3)

When we considered all data (clinical notes, research data for the 10 patients co-enrolled in inpatient studies, and MITS) in Phase 3, only one death certification (case 27) was unchanged compared to pre-Phase 3 coding (**Figures 1–3**). This patient had a single diagnosis (gastroenteritis). The remaining 28 patients either had new diagnoses identified and/or refinements to diagnoses made due to consideration of MITS-derived data (including post-mortem anthropometry). Further, we identified 43 new conditions among 21 (72%) cases in Phase 3, including 22 new causal and 21 new contributing conditions (**Table 1**). At least one new diagnosis was added to the mortality causal chain in 17 cases (59%) and as a contributing condition in 18 cases (62%). Moreover, 26 diagnoses were refined among 19 cases (66%), all of whom had diagnosis adjustments to at least one causal condition.

**Table 1:** New and adjusted diagnoses based on the addition of MITS data compared to clinical and IRS data alone

CoD levels	Counts of new and adjusted clinical diagnoses due to MITS	
	**New diagnoses**	**Adjusted diagnoses**
Part 1: causal conditions (aggregated)	n = 22 among 17 (59%) cases	n = 23 among 19 (66%) cases
Part 2: contributing conditions	n = 21 among 18 (62%) cases	n = 3 among 3 (10%) cases
Total	n = 43 among 21 (72%) cases	n = 26 among 19 (66%) cases
Most frequent diagnoses added and adjusted due to MITS		
*Part 1: causal conditions (aggregated)*	Total n = 22, pneumonia (n = 7, 32%)*, sepsis (n = 5, 23%)†, severe wasting (n = 3, 14%)‡, other (32%)§	Total n = 23, specific pathogens as aetiology of gastroenteritis (n = 8, 35%)¶, specific pathogens as aetiology of pneumonia (n = 6, 26%)║, specific pathogens as aetiology of sepsis (n = 3, 13%)**, severe wasting (n = 3, 13%)††, other (n = 3, 13%)§
*Immediate*	Total n = 12, pneumonia (n = 4, 33%), sepsis (n = 4, 33%), other (n = 4, 33%)§	Total n = 11, specific pathogens as aetiology of gastroenteritis (n = 3, 27%), specific pathogens as aetiology of pneumonia (n = 5, 45%), specific pathogens as aetiology of sepsis (n = 2, 18%), other (n = 1, 9%)§
*Intermediate*	Total n = 6, pneumonia (n = 3, 50%), sepsis (n = 1, (17%), other (n = 2, 33%)§	Total n = 7, specific pathogens as aetiology of gastroenteritis (n = 4, 57%), specific pathogens as aetiology of pneumonia (n = 1, 14%), severe wasting (n = 2, 29%)
*Underlying*	Total n = 4, severe wasting (n = 3, 75%)‡, other (n = 1, 25%)§	Total n = 5, specific pathogens as aetiology of gastroenteritis (n = 1, 20%), specific pathogens as aetiology of sepsis (n = 1, 20%), severe wasting (n = 1, 20%), other (n = 2, 40%)§
*Part 2: contributing conditions*	Total n = 21, stunting (n = 13, 63%), past malaria infection (n = 3, 14%), moderate wasting (n = 2, 10%), other (n = 3, 14%)§	Total n = 3§

CoD – cause of death, IRS – Intensive Research Studies, MITS – minimally invasive tissue sampling

*Due to *Klebsiella pneumoniae* (n = 4), cytomegalovirus (n = 1), *Pneumocystis jirovecii* (n = 1), and *Streptococcus pneumoniae* (n = 1).

†Due to *Klebsiella pneumoniae* (n = 2), *Escherichia coli* (n = 1), *Acinetobacter baumannii* (n = 1), and unidentified pathogen (n = 1).

‡Severe wasting (marasmus) (n = 3).

§Other diagnoses can be found in **Figures 1–3**.

¶Due to enteroaggregative *Escherichia coli* (n = 3), *Salmonella* (n = 2), enteropathogenic *Escherichia coli* (n = 1), rotavirus (n = 1), mixed infection (enteroaggregative *Escherichia coli*, *Campylobacter coli,* and enteropathogenic *Escherichia coli*) (n = 1).

║Due to *Klebsiella pneumoniae* (n = 1), cytomegalovirus (n = 1), *Pneumocystis jirovecii* (n = 2), mixed infection (*Haemophilus influenzae*, *Klebsiella pneumoniae*, *Streptococcus pneumoniae* (n = 1), *Haemophilus influenzae*, *Streptococcus pneumoniae* (n = 1).

**Due to *Klebsiella pneumoniae* (n = 2), methicillin resistant *Staphylococcus aureus* (n = 1)*.*

††Clarified from kwashiorkor to marasmic kwashiorkor (n = 2) and moderate wasting to severe wasting (marasmus) (n = 1).

We coded a total of 142 diagnoses in Phase 3 (which included MITS-derived data), 37% more than in the prior phase (Phase 2 for the 10 co-enrolled patients included inpatient research and clinical data; otherwise, Phase 1 included clinical data only). Specifically, 77 in the causal chain (immediate, intermediate, or underlying) and 65 as contributing conditions. Further, 46 (32%) were undernutrition diagnoses (low birthweight, stunting, moderate wasting, severe wasting, or nutritional oedema). Moreover, 17 (22%) causal chain diagnoses were undernutrition, and 61 (43%) of all diagnoses were infectious conditions, including 46 (60%) in the causal chain. The most common infectious conditions were respiratory infections (n = 18, all with pathogens identified; n = 16 in the causal chain), gastroenteritis (n = 15; n = 9 with pathogens identified, n = 12 in the causal chain), sepsis (n = 9; n = 8 with pathogen identified, all were in the causal chain), HIV infection (n = 7, all in the causal chain), and maternal HIV exposure (n = 6).

In Phase 3, we coded a single causal chain diagnosis in only three cases. As noted above, these are considered immediate and underlying CoDs [21]. The most prevalent underlying CoDs among the 29 cases were severe acute malnutrition (including marasmus and/or kwashiorkor, n = 8), HIV infection (n = 7), and gastroenteritis (n = 3). Fifteen (52%) underlying CoDs were infectious. Further, 24 (83%) immediate CoDs were infectious. Two of the three cases with infectious immediate diagnoses in Phase 1 but not in Phase 3 had those conditions retained but shifted causal pathway locations. The most prevalent immediate CoDs were respiratory infections (n = 11), sepsis (n = 7), and gastroenteritis (n = 6). Of the 65 conditions contributing to mortality, the most prevalent were stunting (n = 20), anaemia (n = 10), maternal HIV exposure (n = 6), low birth weight (n = 5), and moderate wasting (n = 4).

### MITS assessments that contributed to new and adjusted diagnoses

As noted above, 69 new or adjusted causal or contributing diagnoses were added among 28 cases in Phase 3 (**Table 1**). Further, 22 of the new or adjusted diagnoses were undernutrition-related. Children (n = 13) had stunting added as a diagnosis and one was due to the postmortem measurement being consistent with stunting while the antemortem measurement was not, while 12 did not have any antemortem height measurements recorded. Wasting diagnoses (severe (n = 3) and moderate (n = 2)) were added for five cases, all of whom had insufficient anthropometry documentation in their clinical notes. We adjusted one diagnosis of moderate wasting to severe, and one diagnosis of severe wasting was modified to moderate, and in two cases, the form of severe malnutrition was clarified (from kwashiorkor to marasmic kwashiorkor). One of the latter was due to a lower weight-for-length per MITS anthropometry than at admission, the other three lacked any height measurements notations in their clinical notes.

We did not refine non-nutritional non-infectious conditions on the basis of MITS data. However, we added six such conditions among four patients: glomerulonephritis diagnosed based on kidney biopsy HE (n = 1), hepatocyte necrosis with liver failure based on liver biopsy HE (n = 1), one patient with Kaposi sarcoma identified on HE (and confirmed by immunohistochemistry), thrombocytopenia, and pulmonary haemorrhage (based on lung HE), and one case with disseminated intravascular coagulation based on an underlying diagnosis of sepsis and haemorrhage identified on HE of multiple organs.

Further, we identified 19 new and 22 adjusted infectious conditions on the basis of MITS-derived data among 25 cases (**Table 2**; Table S2 in the **Online Supplementary Document**). We identified pathogens for 40 of the 41 (98%) new or adjusted infectious conditions, and 38 (95%) of these pathogens were determined in Phase 3 on the basis of MITS-derived data. Among the 41 new or adjusted infectious diagnoses, custom syndromic TAC panels played a role in identifying 33 specific microbial aetiologies in 33 (81% of these conditions) and, in fact, TAC was necessary for identifying pathogens in 25 (61%).

**Table 2:** New or adjusted infectious disease diagnoses as causes or contributors to mortality based on the addition of MITS data to clinical and IRS data, and the MITS assessments on which the new or adjusted diagnoses were made (presented as n)

Diagnoses	TAC used for diagnosis determination	TAC or special stain used for diagnosis determination*	TAC supported the diagnosis, but it was not necessary for diagnosis determination	Neither TAC nor special stains contributed to the diagnosis determination
Gastroenteritis due to a specific pathogen (n = 9)†	9			
HIV infection (n = 1)‡				1
Malaria (n = 4)§			2	2
Respiratory infection due to a specific pathogen (n = 18)¶	12	5	1║	
Schistosomiasis (n = 1)**				1
Sepsis (n = 1)††				1
Sepsis due to a specific pathogen (n = 7)‡‡	4§§		1¶¶	2║║
Total (n = 41), n (%)	25 (61)	5 (12)	4 (10)	7 (17)

TAC – TaqMan array card

*Histochemical (*i.e.* Gram, Ziehl-Nielsen, Periodic Acid–Schiff, Grocott Methenamine) or immunohistochemical (for CMV, human herpesvirus 8, *Klebsiella pneumoniae, Plasmodium falciparum,* RSV, and Treponema pallidum) stains.

†12 aetiologic pathogens were identified among eight cases. One case with three pathogens (*Campylobacter coli*, enteroaggregative *Escherichia coli*, enteropathogenic *Escherichia coli*, and one case with two pathogens (*Salmonella* and adenovirus) contributed to different parts of the mortality causal chain and therefore counted as two separate diagnoses. Single pathogens included: enteroaggregative *Escherichia coli* (n = 3), enteropathogenic *Escherichia coli* (n = 1), rotavirus (n = 1), and *Salmonella* (n = 1). All pathogens detected on the basis of stool TAC results were supported by clinical presentation.

‡Based on postmortem PCR testing. The TAC did not include an assay for HIV.

§Past malaria infection based on pigment on liver HE (n = 3, including one case with postmortem blood samples positive for *Plasmodium falciparum* by TAC, smear, and RDT). Diagnosis adjustment from cerebral malaria to unspecified severe malaria (n = 1) on basis of brain biopsies negative HE and immunohistochemistry staining for *Plasmodium falciparum*. Postmortem blood TAC, smear, and RDT and antemortem smear and RDT were positive for *Plasmodium falciparum*.

¶Aetiologic pathogens were identified for all 18 respiratory infections included in Phase 3 coding: one case had three pathogens identified as cause of pneumonia (*Haemophilus influenzae*, *Klebsiella pneumoniae*, and *Streptococcus pneumoniae*). Four cases had two pathogens identified as causes of pneumonia (CMV and *Pneumocystis jirovecii*) (n = 2), *Haemophilus influenzae* and *Streptococcus pneumoniae* (n = 1), and CMV and *Klebsiella pneumoniae* (n = 1). For three of these cases, the respiratory infections contributed to different parts of the mortality causal chain and therefore counted as two separate diagnoses. Single pathogens identified included: pneumonia: *Klebsiella pneumoniae* (n = 5), *Streptococcus pneumoniae* (n = 1), and *Pneumocystis jirovecii* (n = 1); bronchiolitis: RSV (n = 1); croup: parainfluenza virus (n = 1); URI: adenovirus (n = 1). For the five cases with multiple pathogens, determinations were all based on TAC (n = 2) and TAC or special stain (Grocott for *Pneumocystis jirovecii* and immunohistochemistry for CMV). Special stains and TAC were assessed on lung tissue with the exception of the parainfluenza virus as a cause of croup identified by TAC from a nasopharyngeal swab.

║*Pneumocystis jirovecii* pneumonia was based on HE and supported by TAC and Grocott stain.

**Based on hepatic HE.

††Negative postmortem CSF microscopy and culture and brain HE shifted diagnosis from antemortem meningitis to sepsis, supported by clinical data.

‡‡All sepsis diagnoses were supported by clinical data, and in five cases by liver HE findings consistent with sepsis.

§§*Klebsiella pneumoniae* (n = 3), including one also with positive postmortem blood culture, and *Escherichia coli* (n = 1) with positive postmortem blood culture.

¶¶Either TAC or postmortem blood culture was considered sufficient evidence for the pathogen (*Acinetobacter baumannii)*.

║║Determined by postmortem blood culture (*Klebsiella pneumoniae* (n = 1), MRSA (n = 1)).

### Level of certainty

We determined the LOC by the DeCoDe panel based on evidence breadth and strength and using specific SOP-based criteria [20]. Based on clinical information only (Phase 1), 49 of 99 causes and contributing conditions (49%) met criteria for LOC level one (certain), while in Phase 3 (based on all data) 113 of the 142 conditions (80%) met LOC level one criteria. In Phase 1, we coded 23 (23%) and 27 (27%) of the 99 diagnoses as LOC two (probable) and three (likely), respectively. In Phase 3, there were 14 (10%) level two and 15 (10%) level three LOCs. When restricted to part one causal conditions only, the LOC determination proportions were not meaningfully different. When restricted to the 10 patients co-enrolled in inpatient studies, the mean LOC in Phase 2 (clinical and IRS data) was greater than for Phase 1 but less than for Phase 3 (Appendix S1 in the **Online Supplementary Document**).

We also examined differences in LOC designation between Phase 3 (all-inclusive data including MITS-related) compared to the previous phase (Phase 2 for the 10 co-enrolled patients and Phase 1 for the 19 patients that were not co-enrolled in inpatient studies) for diagnoses that were similar within the same patient. Across all cases, 92 causal and contributing diagnoses were similar. For three (3%) conditions, the level of certainty decreased from Phase 3 compared to the previous phase. In two of these cases, *Salmonella* was identified as the causative pathogen of gastroenteritis in Phase 3. The LOC reduction was due to DeCoDe SOP higher requirements for coding certainty for some pathogen-specified conditions [20]. For *Salmonella* gastroenteritis, in addition to stool pathogen detection, fever and at least one other illness-related symptom (*e.g.* diarrhoea) are necessary for a LOC one designation. Clinical documentation of fever was absent; therefore, LOC one criteria were not met when the gastroenteritis was ascribed to *Salmonella*. The Phase 3 LOC diminution was also related to an idiosyncrasy in the DeCoDe SOP whereby marasmic kwashiorkor only has a level one or three LOC designation and no level two option. If the criteria for level one for marasmus and kwashiorkor are not met, the SOP calls for defaulting the marasmic kwashiorkor LOC to level three. The updated level three LOC in Phase 3 was due to a clarification of the diagnosis from kwashiorkor (LOC two in Phase 1) to marasmic kwashiorkor in Phase 3 (LOC three). Otherwise, 75 (82%) of the LOCs for the 92 conditions remained the same in Phase 3 compared to the prior phase, while LOC certainty increased for 14 (15%).

## DISCUSSION

This study is the first, that we are aware of, to compare causes and contributors to child deaths by iteratively adding data derived from clinical records, inpatient research with intensive host characterization, and MITS. Anaemia, HIV exposure or infection were identified in half of the cases co-enrolled in inpatient research studies due to systematic HIV and haemoglobin assessments at admission as part of the research protocols, otherwise the clinical-IRS causes and contributing conditions were concordant. However, MITS data led to the vast majority (97%) of cases receiving a new or refined/adjusted diagnosis, including 72% with new conditions identified, and 66% with refined diagnoses. Furthermore, MITS resulted in higher diagnostic level of certainty.

Our study is consistent with another larger study of adults that demonstrated significant diagnostic clinical-MITS discrepancies; with MITS data substantively adding to CoD identification, particularly infection-related deaths [22]. Another study compared malaria as a CoD or contributing condition among inpatients of all ages based on clinical records, verbal autopsy (from clinical record abstracted data), and MITS to gold standard complete diagnostic autopsy and found that while verbal autopsy and clinical records determinations were very insensitive (although highly specific), MITS diagnoses were both highly specific and sensitive [23]. We had not anticipated the degree to which MITS would also add diagnostic precision, especially for the patients who had been co-enrolled in the inpatient studies. Undernutrition (*i.e.* wasting, stunting) diagnoses were frequently added or clarified upon consideration of MITS data, often because anthropometric measurements (along with other standard clinical assessments (*e.g.* HIV status) were not found in clinical notes, either because they were not measured or not documented. Bassat et al. identified malnutrition as the most prevalent underlying CoD among children aged 1–59 months in their large multi-country MITS study and undernutrition anywhere in the causal chain in 24% of cases. Our prevalence was similar despite our small sample size. These data highlight the need for not only individual-based screening for and treatment of undernutrition, but food systems and social determinant-driven approaches – such as maternal education and poverty-alleviation – for prevention [24].

Pneumonia and sepsis were the leading new CoDs, and most with pathogen aetiology that would not be identified without MITS data. Furthermore, pathogen identification – especially for gastroenteritis, pneumonia, and sepsis – represented the leading diagnostic refinements resulting from MITS data. Custom-developed syndromic TACs was the most important data source contributing to both new or adjusted diagnoses.

The prevalence of infectious CoDs observed in our study is consistent with other larger MITS studies [24,25], highlighting *Klebsiella pneumoniae* as an important cause of sepsis and pneumonia [24]. Identification of pathogen burden and prevalence, including among fatal cases, is critically important for identifying emerging infections, shifting disease epidemiology, and directing management guidelines. Many of the pathogens that we identified as causes of sepsis or pneumonia (*e.g. Acinetobacter baumannii* identified by blood TAC and culture, *Klebsiella pneumoniae* by blood or respiratory TAC, *Pneumocystis jirovecii* by respiratory TAC*)* or gastroenteritis (*e.g. Salmonella* by enteric TAC) require broader or alternate antimicrobials compared to first line management according to Malawi national care guidelines (which are based on those from the WHO). Utilising new diagnostic technologies and enhancing implementation of currently available microbiologic testing (*e.g.* blood culture, rapid molecular test) early in inpatient admissions could help improve detection, diagnostic precision, and management. However, many of the MITS laboratory tests for identification of infectious and non-infectious conditions require tissues that are not accessible during life (*e.g.* biopsies of the lung, brain, and liver).

Although the MITS method may be better in detecting infectious CoDs and underperform in the detection of noncommunicable conditions [24], many consequential diagnoses were not suspected in the differential diagnosis prior to examination of MITS data, including non-infectious conditions such as hepatic necrosis as a cause of encephalopathy in a child previously thought to have cerebral malaria based on clinical presentation. In another example, MITS data revealed a child with Kaposi’s sarcoma and secondary thrombocytopenia with pulmonary haemorrhage, diagnosed as an acute respiratory infection based on antemortem data. These much more specific and detailed CoDs are not considered prevalent causes of child mortality. Furthermore, undernutrition was often detected through the MITS procedures. Current CoD estimates are primarily based on verbal autopsy [22], which generally underestimate noncommunicable and malnutrition aetiologies and are unable to make pathogen-specific diagnoses for infectious causes [26]. Postmortem assessments are critical to better understand causes of child mortality to enable further inroads in improving child survival. MITS offers a more feasible and socially acceptable postmortem approach compared to traditional autopsy [5,7–9].

The vast majority (93%) of patients in our study had more than one condition identified as a contributor to or cause of death, and 90% had more than one condition in the causal chain. Currently, the underlying diagnosis in the mortality causal chain is reported as the singular cause of death in proportional mortality statistics which are used in global child health mortality reports and for driving health policy and healthcare resource allocation [21]. This approach assumes that if the underlying cause is addressed, the subsequent cascade would be averted. However, this ignores equally important interruptible life-threatening immediate and intermediate CoDs. For example, the immediate CoDs for eight children in our study with underlying severe wasting represent infections amenable to appropriate, adequate antimicrobial therapeutics. Furthermore, tracking of contributing conditions is important as they are often preventable diagnoses that increase mortality risk (*e.g.* anaemia). Surveillance of contributing conditions could also identify previously unknown predisposing and interruptible links to causes of death.

Consistent with data from the much larger CHAMPS network [24,25], the children in our inpatient mortality study had a complex array of conditions that coalesced to contribute to fatality. The increasing application of postmortem assessments, primarily in the form of MITS due to enhanced feasibility and acceptability compared to traditional autopsy, is demonstrating much more nuanced portrayals of fatality, especially when the full extent of conditions is considered, rather than just traditional approaches focusing solely on underlying aetiologies [24,27]. Tremendous reductions in child mortality have been achieved over the past decades; however, the pace of reduction has slowed. Further reductions in child deaths will necessitate a more holistic approach by focusing on all levels of CoD (immediate, intermediate, underlying) and contributing conditions to inform interventions. Such interventions increasingly need to be individualised, multifaceted, and move away from the ‘one size fits all approach’.

This study is not without limitations. The sample size of 29 hospitalised paediatric cases is small and did not allow for statistical comparisons, although it allowed for an in-depth interrogation at the case level. Queen Elizabeth Central Hospital is a referral centre that receives sick and undernourished children. Due to this factor, the exclusion criteria (*i.e.* known terminal illness, congenital syndrome, injury, or surgical condition) and sample size considerations, the findings may not be generalisable, especially to child deaths in community settings. Differences in cultural views of MITS and social acceptability may also introduce bias and reduce generalisability in MITS studies [28–30]. Variability and bias in interpretation are inherent in the DeCoDe process; however, we took pains to mitigate this through our composition of five multi-disciplinary experts with extensive experience in global child health on the DeCoDe panel and by following SOPs standardised for use in MITS studies [20]. Routine recommended clinical data (*e.g.* anthropometry, complete vital signs, standard HIV and malaria testing for all hospitalised children in this setting) were often not found in clinical notes, and it was impossible to determine if they were not assessed or assessed but not recorded. Nonetheless, other inpatient MITS studies are also confronted with these challenges and highlight the need for documenting in standardised clinical templates rather than blank records without memory prompts [31,32].

Custom-developed syndromic TACs were an important source of pathogen-specific CoD determination and/or confirmation. TAC incorporates multiple real-time PCR pathogen-specific assays into a single test, and the custom-developed TACs in this study significantly increased the number of pathogens screened. PCR is highly sensitive, requiring only small amounts of pathogen nucleic acid, and, unlike traditional culture-based methods, does not require viable organisms [33,34]. However, the high sensitivity of PCR requires adherence to laboratory practices to prevent amplification of contaminating nucleic acids [35]. TAC helps reduce this risk by simplifying the setup with reduced reagent additions since all pathogen-specific oligonucleotides are lyophilised within the closed TAC. We followed TAC methods and interpretation SOPs as used in the much larger CHAMPS MITS studies [24,25]. TAC has proven to be a useful multi-pathogen screening tool for global disease surveillance programs and outbreak responses [34,36]. The high sensitivity of TAC risks overestimation of microbial aetiologies and detection of pathogen nucleic acid in isolation does not definitively indicate disease contribution, and therefore, in the DeCoDe panel process, single sources of microbial data, including TAC results, were never used in isolation, rather always supported by the clinical picture, as well as either histologic or immunohistochemical stains – except gastroenteritis. Standard MITS sampling currently does not include the gastrointestinal tract. We did consider the faecal TAC results in relation to the clinical data and were conservative in our ascertainment of gastrointestinal microbial aetiologies. Our group has demonstrated the feasibility of intestinal sampling [37], and we are exploring the potential role of intestinal TAC, histology, and/or immunohistochemistry to triangulate stool TAC results.

Despite these limitations, strengths of the current study include adherence to international MITS reporting standards [25], newly published guidelines on the ascertainment of wasting and stunting diagnosis in postmortem studies [7], and CoD and LOC standard operation procedures from another large MITS-based surveillance project [20]. Furthermore, we took a novel, in-depth, and layered approach, leveraging data from inpatient research studies with intensive host characterisation, that demonstrated the striking added value of MITS.

## CONCLUSIONS

MITS contributes detail and diagnostic certainty in the determination of causes and contributors to child mortality and, as such, is an important tool to inform strategies such as improved identification of risk factors and implementation of diagnostics for expanded pathogen detection among children hospitalised with acute illness, ultimately averting child deaths. TAC was an important MITS component for illuminating diagnoses and pathogen attribution in our study. Our findings support other recent MITS publications [24,27,38] that suggest moving from a sole focus on the underlying COD to attention to the entire causal chain, which is needed to frame efforts to drive down child mortality in Malawi and other low-resource settings.

## Additional material


Online Supplementary Document
