# Supplementary material for: The contribution of minimally invasive tissue sampling compared to antemortem-derived cause of death determination among inpatient child deaths: the minimally invasive tissue sampling in Malawi study
Source: J Glob Health. 2025 Aug 4;15:04210. doi: 10.7189/jogh.15.04210 (PMC12319396; doi:10.7189/jogh.15.04210)
Supplement: Online Supplementary Document [file jogh-15-04210-s001.pdf]

Supplement to: Voskuijl WP, Chasweka D, Lawrence S, Brals D, Kamiza S, Bandsma R, Berkley JA, Mbale E, Attipa C, Eneya C, Huwa C, Khoswe S, Moxon C, Potani I, Waller JL, Diaz MH, Walson J, Ordi J, Denno DM. The contribution of minimally invasive tissue sampling compared to antemortem-derived cause of death determination among inpatient child deaths: the minimally invasive tissue sampling in Malawi study. J Glob Health. 2025;15:04210.

Table S1.

Pathogen target	Locus or protein encoded	Respiratory TAC	Enteric TAC	Blood/CSF TAC
Acinetobacter baumanii	*OXA-51*	X		X
Adenovirus	*hexon*	X	X	X
Adenovirus 40/41	*fiber protein*		X	
Aeromonas spp.	*glycosyltransferase*		X	
Ascaris lumbricoides	*ITS*		X	
Astrovirus	*capsid*		X	
Bartonella spp.	*ssrA*			X
Bordetella parapertussi, Bordetella bronchiseptica (Insertion sequence IS1001)	*IS1001*	X		
Bordetella pertussis (pertussis toxin)	*ptxSI*	X		
Bordetella pertussis, Bordetella holmseii (Insertion sequence IS481)	*IS481*	X		
*Brucella*spp.	*IS711*			X
Burkholderia pseudomallei	*ttsS*	X		X
C. difficile paLOC	*non-coding IS*		X	
C. difficile tcdA	*tcdA*		X	
C. difficile tcdB	*tcdB*		X	
Campylobacter coli	*cdtB*		X	
Campylobacter jejuni	*hipO*		X	
Candida albicans	*ITS1*			X
Chikungunya virus	*NSP*			X
Chlamydia pneumoniae	*argR*	X		
Chlamydia trachomatis	*tmRNA*	X		
Corynebacterium diptheriae	*rpoB*	X		
Corynebacterium ulcerans, Corynebacterium pseudotuberculosis	*rpoB*	X		
Corynebactrium spp. (tox gene)	*tox*	X		
Coxiella burnettii	*IS1111A*			X
Crimean-Congo Hemorrhagic Fever virus (CCHF)	*N*			X
Cryptococcus neoformans, Cryptococcus gattii	*hypothetical protein*			X
Cryptosporidium parvum	*hypothetical protein*		X	
Cytomegalovirus (CMV)	*glycoprotein B*	X		X
Dengue (pan)	*NSP*			X
Entamoeba histolytica	*18S*		X	

Enterococcus faecalis	*ddl*		X	X
Enterococcus faecium	*ddl*		X	X
Enterovirus	*polyprotein*	X	X	X
Escherichia coli (aatA gene)	*aatA*		X	
Escherichia coli (aaiC gene)	*aaiC*		X	
Escherichia coli (bfpA gene)	*bfpA*		X	
Escherichia coli (eae gene)	*eae*		X	
Escherichia coli (heat-labile enterotoxin)	*LT*		X	
Escherichia coli (heat-stable enterotoxin)	*STh/STp*		X	
Escherichia coli/Shigella	*uidA*			X
Escherichia coli/Shigella (ipaH gene)	*ipaH*		X	
Giardia spp	*18S*		X	
Group A *Streptococcus*	*TetR*	X		X
Group B *Streptococcus*	*cfb*	X		X
Haemophilus influenzae	*hpd3*	X		X
Haemophilus influenzae type B	*bcsB*	X		X
Hepatitis E virus	*capsid protein*			X
Herpes simplex virus 1 (HSV1)	*envelope glycoprotein G*			X
Herpes simplex virus 2 (HSV2)	*virion glycoprotein D*			X
Human coronavirus 1 (229E)	*N*	X		
Human coronavirus 2 (NL63)	*N*	X		
Human coronavirus 3 (OC43)	*N*	X		
Human coronavirus 4 (HKU1)	*RdRp*	X		
Human metapneumovirus (HMPV)	*Fusion protein*	X		
Influenza A	*M*	X		
Influenza B	*NEP*	X		
Japanese encephalitis virus	*polyprotein*			X
Klebsiella pneumoniae	*DC*	X		X
Lassa Fever virus target 1 (lineage 4)	*S segment*			X
Lassa Fever virus target 2 (lineage 1 and 2)	*S segment*			X
Leptospira (pan-serovar)	*lipL32*			X
Listeria monocytogenes	*prfA*			X
Measles	*N*	X		X
MERS coronavirus (N gene)	*N*	X		
MERS coronavirus (upE gene)	*UpE*	X		
Moraxella catarrhalis	*PurH*	X		

Mumps	*N*			X
Mycobacterium tuberculosis	*IS6110*	X	X	X
Mycoplasma pneumoniae	*CARDS Tx*	X		
Neisseria gonorrhoeae	*porA*			X
Neisseria meningitidis	*sodC*			X
Nipah virus	*N*			X
Norovirus GI	*ORF 1-2*		X	
Norovirus GII	*ORF 1-2*		X	
Orientia tsutsugamushi	*htrA*			X
PAN Lassa Fever virus (all lineages)	*L segment*			X
Parainfluenza virus type 1	*HN*	X		
Parainfluenza virus type 2	*N*	X		
Parainfluenza virus type 3	*HN*	X		
Parainfluenza virus type 4	*N*	X		
Parechovirus	*polyprotein*			X
Parvovirus B19	*VP2 capsid protein*			X
Plasmodium falciparum	*18S*			X
Plasmodium vivax	*18S*			X
Pneumocystis jirovecii	*DHPS*	X		
Pseudomonas aeruginosa	*gyrB*	X		X
Respiratory syncytical virus (RSV)	*M*	X		
Rhinovirus	*polyprotein*	X		
Rickettsia spp.	*50S*			X
Rift Valley Fever	*L*			X
Rotavirus A	*NSP3*		X	
Rotavirus B	*NSP2*		X	
Rotavirus C	*VP6*		X	
Rotavirus non-typable	*NSP2*		X	
Rubella	*NSP*	X		X
Salmonella paratyphi A	*hypothetical protein*			X
Salmonella spp.	*ttrRSBCA*		X	X
Salmonella typhi	*lpfA*			X
Sapovirus I/II/IV	*RdRp*		X	
Sapovirus V	*RdRp*		X	
Shiga toxin/Shiga-like toxin 1	*stx1*		X	
Shiga-like toxin 2	*stx2*		X	
Staphylococcus aureus	*hypothetical protein*	X		X
Streptococcus pneumoniae	*lytA*	X		X
Streptococcus suis	*fbp*			X

Toxoplasma gondii	*18S*			X
Treponema pallidum	*polA*			X
Trichuris trichuria	*18S*		X	
Ureaplasma spp.	*URE*			X
Varicella zoster virus	*ORF62*	X		X
Vibrio cholerae	*ompW*		X	
Vibrio cholerae toxigenic	*ctxA*		X	
West Nile Virus	*polyprotein*			X
Yellow Fever virus	*polyprotein*			X
Yersinia spp.	*pal*		X	X
Zika	*polyprotein*			X

Abbreviations: CSF – cerebrospinal fluid, TAC – TaqMan array card

Table S2. New or adjusted infectious disease diagnoses as causes or contributors to mortality based on the addition of MITS data to clinical and IRS data, and the MITSassessments on which the new or adjusted diagnosis were made. This is a version of Table 2 withexpanded footnotes.

	TAC necessary for diagnosis determination	TAC or special stain* necessary for diagnosis determination	TAC supported the diagnosis but was not necessary for diagnosis determination	Neither TAC nor special stains contributed to diagnosis determination
Gastroenteritis due to a specific pathogen (n=9)†	9			
HIV infection (n=1)‡				1
Malaria (n=4)§			2	2
Respiratory infection due to a specific pathogen (n=18)¶	12	5	1‖	
Schistosomiasis (n=1)**				1
Sepsis, NOS (n=1)††				1
Sepsis due to a specific pathogen (n=7)‡‡	4		1	2
**Total (n=41)**	**25 (%)**	**5 (%)**	**4 (%)**	**7 (%)**

* H&E was not considered a “special stain”. Special stains included Ancillary histochemical (i.e., Gram, Ziehl-Nielsen, Periodic Acid–Schif, Grocott Methenamine) and immunohistochemical stains (for cytomegalovirus, human herpesvirus 8, *Klebsiella pneumoniae, Plasmodium falciparum,* respiratory syncytial virus, and Treponema pallidum).

† For 5 of these gastroenteritis diagnoses, gastroenteritis was also identified as a diagnosis in Phase 1 – i.e., prior to assessment of MITS-derived data, but without a specific identifiable pathogen. 12 etiologic pathogens were identified among 8 cases. 1 case with 3 pathogens (*Campylobacter coli*, enteroaggregative *E. coli* (EAEC)*,* enteropathogenic *E. coli* (EPEC)) and 1 case with 2 pathogens (*Salmonella* and adenovirus) listed in different parts of the mortality causal chain and therefore counted as two separate diagnoses. Single pathogens included: EAEC (n=3), EPEC (n=1), rotavirus (n=1), and *Salmonella* (n=1). All pathogens detected on the basis of stool TAC results were supported by clinical presentation.

‡ Based on PM PCR testing. The TAC cards did not have a target for HIV.

§ Past malaria infection on the basis of pigment on liver H&E (n=3, including one case with PM blood samples positive for *P. falciparum* by TAC, smear, and RDT). Diagnosis adjustment from cerebral malaria to unspecified severe malaria (n=1) on basis of brain biopsies negative H&E and IHC staining for *P. falciparum;* PM blood TAC, smear, and RDT and antemortem smear and RDT were positive for *P. falciparum*..

¶ Diagnoses included pneumonia (n=15), bronchiolitis (n=1), upper respiratory infection (URI) (n=1), and acute obstructive laryngitis/croup (n=1).

For 10 of these respiratory infection diagnoses, respiratory infections was also identified as a diagnosis in Phase 1 – i.e., prior to assessment of MITS-derived data, but without a specific identifiable pathogen, while 8 respiratory infection diagnoses were not identified prior to evaluation of MITS-derived data. For these 8 respiratory infections newly identified based on MITS-derived data, the following data identified the diagnosis in conjunction with the clinical presentation and other MITS-derived data (lung H&E with or without lung gram stain or IHC): TAC results (n=6), or TAC or IHC results (n=1), TAC and IHC results (n=1).

Etiologic pathogens were identified for all 18 respiratory infections: 1 case had 3 pathogens identified as cause of pneumonia (*Haemophilus influenzae*, *K. pneumoniae*, and *Streptococcus pneumoniae*); 4 cases

had 2 pathogens identified as causes of pneumonia (CMV and *Pneumocystis jirovecii*) (n=2), *H. influenzae* and *S. pneumoniae* (n=1), and CMV and *K. pneumoniae* (n=1); for 3 of these cases, the respiratory infections contributed to different parts of the mortality causal chain and therefore counted as two separate diagnoses. Single pathogens identified included: pneumonia: *K. pneumoniae* (n=5), *S. pneumoniae* (n=1), and *P. jirovecii* (n=1); bronchiolitis: RSV (n=1); croup: parainfluenza virus (n=1); URI: adenovirus (n=1). Special stains and TAC were assessed on lung tissue with the exception of the parainfluenza virus serogroup 3 as a cause of croup, which was identified by TAC from a nasopharyngeal swab.

‖ Diagnosis of *P. jirovecii* pneumonia was based on H&E (severe diffuse alveolar damage characteristic of *P. jirovecii*) and supported by TAC and Grocott stain. (For two other *P. jirovecii* pneumonia diagnoses the H&E supported the diagnosis but diffuse alveolar damage was mild or equivocal and TAC or Grocott stain was needed to make the diagnosis.)

** Based on hepatic H&E

†† Pre-consideration of MITS-derived data, meningitis was thought to be the immediate cause of death based on clinical presentation. Antemortem lumbar puncture was not performed. PM cerebrospinal fluid (CSF) microscopy and brain H&E was within normal limits and CSF culture was negative. Hence in Phase 3 sepsis was considered the most likely CoD based on clinical data, although no definitive pathogen could be determined as etiologic.

‡‡ In the 7 cases with pathogens identified as etiology for sepsis, sepsis was supported by clinical data and in 5 cases also by liver H&E findings consistent with sepsis. The case with wounds secondary to burns and methicillin resistant *Staphylococcus aureus* identified on blood culture had a normal liver biopsy (other than mild chronic portal inflammation). The case with *E. coli* sepsis was determined on the basis of PM blood culture and TAC results, and did have severe hepatic necrosis but this was determined to be unrelated to sepsis. The *Acinetobacter baumannii* sepsis diagnosis was based on positive PM blood culture or TAC results. The remaining 4 cases were diagnosed with *K. pneumoniae* sepsis, one by PM blood culture and 3 by TAC (including one that also had a positive PM blood culture).

Abbreviations: CMV – cytomegalovirus, CSF – cerebrospinal fluid, GS – Gram stain, H&E – haematoxylin and eosin, IRS – intensive research studies, MITS – minimally invasive tissue sampling, MRSA – methicillin resistant Staphylococcus aureus, PM – postmortem, RSV – respiratory syncytial virus, TAC – TaqMan array card, URI – upper respiratory infection

Appendix S1. Level of Certainty (LOC) determinations for the 10 patients co-enrolled in inpatient studies

Based on clinical information only (Phase 1), 24 of 36 of causes and contributors to mortality (67%) met criteria for LOC level 1 (certain). Based on clinical and IRS data (Phase 2), 33 of the 41 (80%) conditions met LOC level 1 criteria. Lastly, based on clinical, IRS, and MITS data (Phase 3) 47 of the 56 conditions (84%) met LOC level 1 criteria. In Phase 1, 8% and 25% of the 36 diagnoses were coded as LOC 2 (probable) and 3 (likely), respectively. In Phase 2, 7% and 12% of the 41 were coded as LOC 2 and 3, respectively. In Phase 3, there were only 1 (2%) LOC level 2 and 8 (14%) LOC level 3 diagnoses of the 56.

The mean LOC in Phases 1, 2, and 3 were 1.58 (standard deviation (SD) = 0.87), 1.31 (SD = 0.69), and 1.13 (SD = 0.72), respectively.

	Phase 1<br>Clinical only	Phase 2<br>Clinical + IRS	Phase 3<br>Clinical + IRS + MITS
Causes and contributors to mortality N	36	41	56
LOC 1 N (%)	24 (67%)	33 (80%)	47 (84%)
LOC 2 N (%)	3 (8%)	3 (7%)	1 (2%)
LOC 3 N (%)	9 (25%)	5 (12%)	8 (14%)
Mean LOC	1.58	1.31	1.13
